# Low-Density Lipoprotein Receptor-Related Protein 1 (LRP1) as a Novel Regulator of Early Astroglial Differentiation

**DOI:** 10.3389/fncel.2021.642521

**Published:** 2021-02-18

**Authors:** Ramona Romeo, Damian Boden-El Mourabit, Anja Scheller, Melanie D. Mark, Andreas Faissner

**Affiliations:** ^1^Department of Cell Morphology and Molecular Neurobiology, Ruhr-University Bochum, Bochum, Germany; ^2^Behavioral Neuroscience, Ruhr-University Bochum, Bochum, Germany; ^3^Department of Molecular Physiology, Center for Integrative Physiology and Molecular Medicine (CIPMM), University of Saarland, Homburg, Germany

**Keywords:** astrocyte heterogeneity, differentiation, astrocyte functions, hippocampus, *in vivo* knockout model, LRP1

## Abstract

Astrocytes are the most abundant cell type within the central nervous system (CNS) with various functions. Furthermore, astrocytes show a regional and developmental heterogeneity traceable with specific markers. In this study, the influence of the low-density lipoprotein receptor-related protein 1 (LRP1) on astrocytic maturation within the hippocampus was analyzed during development. Previous studies mostly focused on the involvement of LRP1 in the neuronal compartment, where the deletion caused hyperactivity and motor dysfunctions in knockout animals. However, the influence of LRP1 on glia cells is less intensively investigated. Therefore, we used a newly generated mouse model, where LRP1 is specifically deleted from GLAST-positive astrocytes co-localized with the expression of the reporter tdTomato to visualize recombination and knockout events *in vivo*. The influence of LRP1 on the maturation of hippocampal astrocytes was assessed with immunohistochemical stainings against stage-specific markers as well as on mRNA level with RT-PCR analysis. The examination revealed that the knockout induction caused a significantly decreased number of mature astrocytes at an early developmental timepoint compared to control animals. Additionally, the delayed maturation of astrocytes also caused a reduced activity of neurons within the hippocampus. As previous studies showed that the glial specification and maturation of astrocytes is dependent on the signaling cascades Ras/Raf/MEK/Erk and PI3K/Akt, the phosphorylation of the signaling molecules Erk1/2 and Akt was analyzed. The hippocampal tissue of LRP1-deficient animals at P21 showed a significantly decreased amount of activated Erk in comparison to control tissue leading to the conclusion that the activation of this signaling cascade is dependent on LRP1 in astrocytes, which in turn is necessary for proper maturation of astrocytes. Our results showed that the deletion of LRP1 at an early developmental timepoint caused a delayed maturation of astrocytes in the hippocampus based on an altered activation of the Ras/Raf/MEK/Erk signaling pathway. However, with ongoing development these effects were compensated and the number of mature astrocytes was comparable as well as the activity of neurons. Therefore, LRP1 acts as an early regulator of the differentiation and maturation of astrocytes within the hippocampus.

## Introduction

Astrocytes are the most abundant cell type within the central nervous system (CNS) and were formerly known as passive players supplying neurons with energy substrates and providing structural support (Yong et al., 1998; Gordon et al., 2007; Iadecola and Nedergaard, 2007; Sofroniew and Vinters, 2010). However, more recent studies prove that astrocytes are actively contributing to the neuronal transmission in forming the tripartite synapse with pre- and postsynapse (Bushong et al., 2004; Freeman, 2010; Savtchouk and Volterra, 2018). The majority of astrocytes are generated postnatally after the generation of neurons (Kriegstein and Alvarez-Buylla, 2009). The postnatal and terminal differentiation into astrocytes seem to be dependent on intracellular signaling cues (Qian et al., 2000; Shen et al., 2006). Previous studies have shown that the glial specification is influenced by the activation of the Ras/Raf/MEK/Erk pathway (Franzdóttir et al., 2009; Li et al., 2012). The activity of these signaling pathways is regulated by the formation of cell surface receptor complexes causing intracellular signaling cascades. One known activator of the Ras/Raf/MEK/Erk pathway is the complex formation between the low-density lipoprotein receptor-related protein 1 (LRP1) with the platelet-derived growth factor receptor β (PDGFRβ) (Boucher et al., 2002; Loukinova et al., 2002) or tropomyosin receptor kinase A (trkA) (Spuch et al., 2012). Furthermore, the association of LRP1 with PDGFRβ also influences the activation of the PI3K/Akt pathway, another important signaling cascade involved in glia specification (Muratoglu et al., 2010).

LRP1 is a member of the low-density lipoprotein receptor (LDL) superfamily and is also known as CD91 or α2-macroglobulin receptor (Binder et al., 2000; Liu et al., 2000; Marschang et al., 2004). The receptor is ubiquitously expressed but enriched in liver, blood vessels and the CNS (Lillis et al., 2005). The receptor consists of an extracellular, 515 kDa α-chain and an intracellular, 85 kDa β-chain, non-covalently bound on the cell surface (Lillis et al., 2005). Over 40 potential ligands are known for LRP1 and ligand binding to LRP1 leads to endocytosis of the whole complex, making LRP1 formerly known for its endocytic function (Lillis et al., 2005; Bres and Faissner, 2019). In addition, LRP1 can form complexes with other receptors leading to the activation of intracellular pathways. Thereby, LRP1 was unraveled as a regulator for different cellular processes, such as proliferation (Zhang and Liu, 2002), differentiation (Seger and Krebs, 1995; Ortega and Alcántara, 2010; Safina et al., 2016) and apoptosis (Burris, 2013; Kumar et al., 2013). Total loss of LRP1 within the whole organism leads to embryonic lethality, highlighting the importance of LRP1 (Herz et al., 1992, 1993). However, the influence of LRP1 in regard to astrocyte function was not intensively studied. The deletion of LRP1 in astrocytes caused a negatively influenced cellular uptake of Aβ and degradation involved in Alzheimer’s Disease (AD) progression (Liu et al., 2017). The deletion of LRP1 from radial glia cells resulted in altered numbers of astroglial subpopulations *in vivo* (Bres et al., 2020). Furthermore, we investigated the influence of LRP1 on astrocytes *in vitro*, where we observed that the knockout of LRP1 in primary cortical astrocytes caused an altered expression of mature astrocytic markers. Additionally, the functionality of astrocytes in regard to the neuronal transmission as well as on synaptogenesis of hippocampal neurons was negatively influenced (Romeo et al., 2021).

Based on the findings and preliminary data we decided to investigate the influence of LRP1 on the maturation and functions of hippocampal astrocytes *in vivo*. Therefore, we used our newly generated mouse model (Romeo et al., 2021), where LRP1 is specifically deleted from GLAST-positive astrocytes via the intraperitoneal injection of Tamoxifen into lactating mothers causing the loss of LRP1 in their litters. Via the expression of the reporter tdTomato, we were able to visualize recombination events as well as the deletion of LRP1. The maturation of astrocytes in LRP1-deficient tissue was assessed via immunohistochemical stainings as well as with RT-PCR. Additionally, we wanted to further characterize our knockout model and performed behavioral tests to investigate the motor coordination and cognitive abilities.

## Materials and Methods

### Animals

As previously described (Romeo et al., 2021), we generated a new triple transgenic mouse model where *Lrp1* was specifically deleted from GLAST-positive astroglial precursor cells by using the GLAST::CreERT2-mouse line (Mori et al., 2006) bred with the LRP1^flox/flox^-mouse line (Rohlmann et al., 1996). For the mating of control, respectively, knockout animals, animals with a heterozygous expression of the Cre recombinase under the GLAST promotor were used (Romeo et al., 2021). Therefore, not all pups of the litters were suitable for the study as some expressed the Cre recombinase homozygously or not at all. Additionally, the application of Tamoxifen to lactating mothers caused mortality of the pups. This study was performed in approval with the State Agency for Nature, Environment and Consumer Protection Northrhine-Westphalia (Landesamt fuer Umweltschutz, Naturschutz und Verbraucherschutz; file number: 84-02.04.2016.A482). The animals were housed with a 12 h light/dark cycle and access to food and water *ad libitum*. To induce the knockout in the new born pups lactating mothers received 100 mg/kg of bodyweight of a 10 mg/ml stock solution of Tamoxifen (TAM; Sigma Aldrich, Chemie GmbH, Munich, Germany, Catalog-No.: t5648) in corn oil 5 days after birth. TAM was applied for 5 days in a row in accordance to Jahn et al. (2018). The genotyping of the animals was performed as described in Romeo et al. (2021). Briefly, tail biopsies were taken and lysed with 200 μl of *DirectPCR^®^ Lysis Reagent Tail* (Peqlab, VWR Life Science, Radnor, PA) and 0.2 mg/ml Proteinase-K (Sigma-Aldrich, Chemie GmbH, Munich, Germany) at 55°C at 350 rpm overnight. The lysis was stopped by heating the samples to 85°C for 45 min at 350 rpm. For the genotyping, gene-specific primers were used.

### Transcardial Perfusion of Animals

For the investigation of the influence of LRP1 on the maturation of astrocytes and their functionality, three developmental stages were analyzed (P21, P28, and P56-P70, summarized as adult). At the stage-specific age either control or knockout animals were anesthetized with 100 mg/kg ketamine (CP-Pharma, Burgdorf, Germany), 10 mg/kg Xylazin (CP-Pharma) in 0.9% NaCl. Afterward, the animals were transcardially perfused with phosphate buffered saline (PBS; 137 mM sodium chloride, 3 mM potassium chloride, 6.5 mM disodium hydrogen phosphate, 1.5 mM potassium dihydrogen phosphate; pH 7.3) and subsequently with 4% (w/v) paraformaldehyde (PFA) when the tissue was used for immunohistochemistry. Tissue for the RNA and protein isolation was prepared and transferred to a dish containing PBS. Then the hippocampus was removed and frozen in liquid nitrogen until further use.

### Immunohistochemistry

Prepared tissue was post-fixed in 4% (w/v) PFA for additional 6 h and then dehydrated in a sucrose gradient (10%, 20%, 30% (w/v) sucrose in PBS). Afterward, the tissue was embedded in Tissue Freezing Medium (Leica, Wetzlar, Germany) at −80°C until further use. Cryosections of 14 μm were prepared. The cryosections were stained as previously described (Schäfer et al., 2019; Ulc et al., 2019). Shortly, cryosections were transferred to citric acid buffer (solution A: 0.1 M citric acid-1-hydrate, solution B: 0.1 M Na-citrate-dihydrate; 1 mM of solution A and 4 mM of solution B in *aqua dest*.) for 1 h at 70°C. After cooling and blocking (10% (v/v) normal goat serum (NGS; Jackson Immuno Research Labs, Catalog-No.: 005-00-121; AB_2336990) in PBT-1 [10% (v/v) BSA, 0.1% (v/v) Triton-X 100 in PBS)] for 1 h, primary antibodies were diluted in blocking solution and sections were incubated over night at 4°C. For our analysis we used the following primary antibodies: LRP1 (1:500, Abcam, Catalog-No.: ab92544; AB_2234877), GFAP (1:300, DAKO, Catalog-No.: Z0334; AB_10013382), GLT-1 (EAAT2; 1:100, Santa Cruz, Catalog-No.: sc-365634; AB_10844832), S100 (1:750, Sigma-Aldrich, Catalog-No.: ab868; AB_306716), c-Fos (1:500, Abcam, Catalog-No.: ab208942; AB_2747772), CC1 (APC; 1:100, Abcam, Catalog-No.: ab16794; AB_443473), NeuN (1:500, Millipore, Catalog-No: MAB377; AB_2298772) and phospho-Histone H3 (PH3; 1:300, Millipore, Catalog-No.: 06-570; AB_310177). Further on, sections were washed thrice with PBS for 20 min, followed by incubation with appropriate secondary antibodies diluted in PBS/A (0.1% bovine serum albumin (w/v; BSA; Sigma-Aldrich, Catalog-No.: A7030) in PBS) for 2 h at room temperature. We used the following secondary antibodies coupled with Cy2: Goat anti-mouse Cy2, Jackson Immuno Research Labs, Catalog-No.: 115-545-044; AB_2338844 and Goat anti-rabbit Cy2, Jackson Immuno Research Labs, Catalog-No.: 111-545-045; AB_2338049 (1:250) and the cell nuclei marker Hoechst (1:100). Further on, the washing steps with PBS were repeated in the dark and the sections were covered with Immu-Mount (Thermo Fisher Scientific, Waltham, Massachusetts, United States; Catalog-No.: 9990402). Images were taken with the AxioZoom by Zeiss (Oberkochen, Germany).

### RNA Isolation, cDNA Synthesis and Real-Time PCR Analysis

One hippocampal half of animals was lysed with lysis buffer of the Gene Elute Mammalian Total RNA Miniprep Kit (Sigma Aldrich; Catalog-No.: RTN350-1KT) and RNA was isolated according to manufacturer’s manual. To synthesize cDNA, 1 μg of RNA was used in accordance to the First Strand cDNA-synthesis Kit (Fermentas, Waltham, MA, United States; Catalog-No.: K1612). To investigate the maturation of astrocytes within astrocyte-specific LRP1-depleted hippocampi, stage-specific astroglial markers were used (see Table 1) with *ß-actin* as reference gene.

**TABLE 1:** Investigated genes in the expression profile analysis of hippocampal tissue of either LRP1-deficient animals compared to control animals *in vivo*.

Gene	Primer sequence	GenBank no.
*β-actin*	F: 5′-TATGCCAACACAGTGCTGTCTGGTGG-3′ R: 5′-TAGAAGCATTTGCGGTGGACAATGG-3′	NM_007393.5
*Akt1*	F: 5′-5′-CGACGTAGCCATTGTGAAGG-3′ R: 5′-CTTCCTGCCTCTTGAGTCCA-3′	NM_009652.3
*Aldh1l1*	F: 5′-GGAAGTTGAGAGGGGAGGAC-3′ R: 5′-GGAAGTTGAGAGGGGAGGAC-3′	BC030730.1
*Aquaporin-4*	F: 5′-TTGCTTTGGATCAGCATTG-3′ R: 5′-TGAGCTCCACATCAGGACAG-3′	NM_009700.3
*Fgfr3*	F: 5′-GTCCTGTTCTGGCCAATGTT-3′ R: 5′ -GTTTCTGGCAGCCAAGTCTC-3′	NM_001205270.1
*Gfap*	F: 5′-CGACTATCGCCGCCAACTGC-3′ R: 5′-GCGATCTCGATGTCCAGGGCT-3′	NM_001131020.1
*Glast*	F: 5′-GGCGGCCCTAGATAGTAAGG-3′ R: 5′-AGAGTCTCCATGGCCTCTGA-3′	XM_021208184.2
*Glt-1*	F: 5′-ATGATCATGTGGTACTCCCCTC-3′ R: 5′-TTGTCGTCGTAAATGGACTGC-3′	NM_001077514.4
*Gria1*	F: 5′-CCGTTGACACATCCAATCAG-3′ R: 5′-GTTGGCGAGGATGTAGTGGT-3′	NM_001113325.2
*Gria2*	F: 5′-AACGGCGTGTAATCCTTGAC-3′ R: 5′-CTCCTGCATTTCCTCTCCTG-3′	NM_013540.3
*Grin1*	F: 5′-CGGCTCTTGGAAGATACAGC-3′ R: 5′-TTGTAGACGCGCATCATCTC-3′	NM_008169.3
*Grin2a*	F: 5′-GCTGTCAGCACTGAATCCAA-3′ R: 5′-ATCCCTGGGAGAACTTGCTT-3′	NM_008170.3
*Grin2b*	F: 5′-GTGAGAGCTCCTTTGCCAAC-3′ R: 5′-GGGTTGGACTGGTTCCCTAT-3′	NM_008171.3
*Lrp1*	F: 5′-GGTAGTTGTTTCCTCAATGCTC-3′ R: 5′-TGTTGCTGACTAACAACCTGCT-3′	NM_008512.2
*Lrp2*	F: 5′-CTTCTGATGAGTCCGCTTGC-3′ R: 5′-AGTTCCCATTGCTGCACTTG-3′	NM_001081088.2
*Mtor*	F: 5′-CTTGCTGATCCTCAACGAGC-3′ R: 5′-CTGGATCAGCGAGTTCTTGC-3′	NM_020009.2
*Nestin*	F: 5′-CTCGAGCAGGAAGTGGTAGG-3′ R: 5′-GTTAGCGCTGCCRCRAGACC-3′	NM_016701.3
*S100*	F: 5′-TGTCTTCCACCAGTACTCCG-3′ R: 5′-ACTCCTGGAAGTCACACTCC-3′	NM_009115.3

*The used primers were self-designed with the according nucleotide sequence (see GenBank No.).*

### Sample Synthesis and Western Blotting

The other hippocampal half was lysed with radioimmunoprecipitation assay buffer (RIPA; 10 mM Tris-HCl (pH 8.0), 1 mM ethylenediaminetetraacetic acid (EDTA), 0.5 mM ethylene glycol-bis(β-aminoethyl ether)-N,N,N’,N’-tetraacetic acid (EGTA), 1% (v/v) Triton X-100, 0.1% (w/v) sodium deoxycholate, 0.1% (v/v) sodium dodecyl sulphate (SDS), 140 mM NaCl, Sigma) with 1% (v/v) of protease inhibitors phenylmethylsulfonyl fluoride (PMSF) and 1% (v/v) aprotinin (APR). The tissue was manually lysed and further on triturated with pipettes to dissolve the tissue. Protein concentration was measured with the Pierce^TM^ BCA^TM^ Protein Assay (Thermo Fisher Scientific). For the western blot analysis 30 μg of protein was applied to a 12% sodium dodecyl sulfate polyacrylamide (SDS) gel as described in Hennen et al. (2011). We used following primary antibodies: LRP1 (1:10,000, Abcam, Catalog-No.: ab92544; AB_2234877), pAkt (1:5,000, Cell Signaling Technologies, Catalog-No.: #4,060, AB_2315049), tAkt (1:5,000; Cell Signaling Technologies, Catalog-No.: #4,691, AB_915783), pErk1/2 (1:5,000; Santa Cruz, Catalog-No.: sc-7383, AB_627545), tErk1/2 (1:5,000; Santa Cruz, Catalog-No.: sc-514302, AB_2571739), GLT-1 (EAAT2; 1:200, Santa Cruz, Catalog-No.: sc-365634; AB_2571739) and α-tubulin (1:5,000, Sigma Aldrich, Catalog-No.: T9026, AB_477593), used as control and for normalization. The protein expression was detected with the Clarity^TM^ Western ECL Substrate by BioRad (Feldkirchen, Germany) and visualized with the MicroChemie chemiluminescence device with the Gel Capture Software by biostep (Burkhardtsdorf, Germany; Version: 6.6).

### Motor and Cognitive Behavior Tests

To investigate if the absence of astroglial LRP1 has an influence on the behavior or motor coordination, we used six conditional knockout animals (GLAST^CreERT2/wt^Rosa26^fl/fl^LRP1^fl/fl^; 4 males, 2 females) and five control animals (GLAST^CreERT2/wt^Rosa26^fl/fl^LRP1^wt/wt^; 4 males, 1 female) at the age of 2 months, for all tests. The rotarod test assessed the motor coordination as well as the balance of the knockout animals in comparison to the control animals as described (Mark et al., 2011; Maejima et al., 2013). Briefly, after acclimation to the rotarod device (Columbus Instruments) with four rotations per minute (rpm) for 1 min the rod was accelerated at 0.1 rpm/s up to 40 rpm. Speed and latency to fall were recorded and averaged from three trials per mouse. To test for fine motor coordination mice were tested on the beam walk. Mice were placed on a horizontally 70 cm long beam (1 cm wide and 60 cm above the table surface) with a 20 cm^2^ goal box. The animals underwent 2 days of training (six trials per day) before the data was collected. The time to transverse the beam, idle time (immobile time at start), the number of slips of the right and left hindlimbs and falls were investigated. Animals were given a maximum of 120 s to reach the goal box or for a fall. The collected data was averaged over three trials per mouse. The muscle strength of the mice was evaluated with the hangwire test. Mice were placed upside down on a wire screen (1.2 × 1.2 cm) 50 cm above a cage and the latency to fall was recorded. Another test to investigate motor coordination and balance is the vertical pole test. Here, the mice were placed on a 50 cm vertical metal pole (1 cm diameter) facing upwards. The latency to descend down to the cage was recorded. When the animals fell down or slid down the pole rear end first, the maximum of 120 s was given. To assess the cognitive abilities that are related to memory and learning processes the 2-object novel object recognition test (NOR) based on the established protocol (Lueptow, 2017) was used. Briefly, the test consisted of 3 days, habituation to the arena (50 × 50 cm), testing to two similar objects and testing to one familiar and one novel object. The latency to investigate the new object in comparison to the familiar one was recorded and defined as the preference index. Cognitive functions of the mice were also investigated using the T-maze test (Deacon and Rawlins, 2006). Briefly, mice were placed in a T-shaped arena and explored the left or right arm of the T. The mice chose one arm based on curiosity. When the mouse entered one of the arms in the T a door at the start of the arm was closed to allow 30 s exploration. Then the mice were again placed at the start of the arena and were allowed to choose one arm. Unperturbed animals would explore the previously unknown arm of the arena, whereas impaired animals were not able to remember which part was already explored. Five trials per animal were averaged and the alternation value was evaluated.

For the statistical analyses, we firstly evaluated if the data was normally distributed with the Shapiro-Wilk-Normality test. As the data was normally distributed the Student’s *t*-test was used to investigate the level of significance (^∗^*p* ≤ 0.05, ^∗∗^*p* ≤ 0.01, ^∗∗∗^*p* ≤ 0.001).

### Statistical Analyses

The immunohistochemical stainings were investigated with the cell counter plug-in by Image J. Here, all tdTomato-positive cells were quantified in the spaces between the *Cornu Ammonis* layers of the hippocampus to provide the most accurate quantification of recombined cells (see Supplementary Figure 1). Afterward, all marker-specific and tdTomato-double positive cells were quantified to evaluate specificity of recombination. Exemplary double-positive cells, meaning tdTomato- and marker-positive cells, were highlighted with arrowheads in the figures. Additionally, the nuclei within the molecular layers of the hippocampus were evaluated to investigate the number of recombined cells. Intensity of the bands of the PCR and western blot analyzes were also measured with ImageJ. The band intensities were normalized to the expression of β-actin, respectively, α-tubulin. The results are shown as the mean ± the standard error of the mean (SEM). The endpoint-PCR analysis was used as a semi-quantitative method to analyze potentially differentially expressed genes after knockout indication. The cycle number for the RT-PCR were below the amplificate saturation level. The depicted PCR results are only shown as an example. For the statistical analyses, we firstly evaluated if the data was normally distributed with the Shapiro-Wilk-Normality test. As the data was normally distributed, we used the two-way ANOVA with *post-hoc* Bonferroni test to analyze the level of significance (^∗^*p* ≤ 0.05, ^∗∗^*p* ≤ 0.01, ^∗∗∗^*p* ≤ 0.001).

## Results

### Successful Deletion of LRP1 in Hippocampal Astrocytes

**FIGURE 1:**
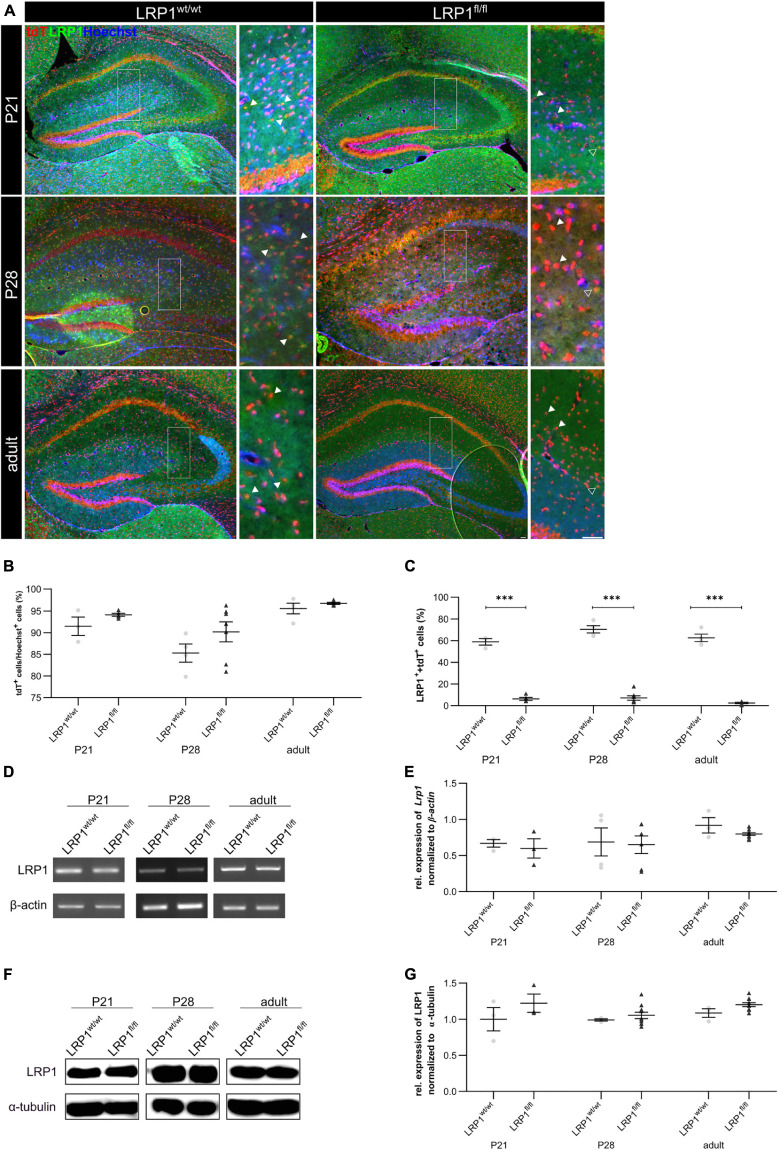
Knockout induction via lactating mothers was successful *in vivo*. The immunohistochemical staining against LRP1 (green) and tdTomato (red) revealed the expression of LRP1 by recombined astrocytes during development indicated by the arrowhead **(A)**. The recombination rate differed between 85 and 97% during development in LRP1-deficient and control hippocampi **(B)**. The knockout efficiency was evaluated by the quantification of LRP1- and tdTomato-double positive cells divided by the total number of recombined cells within the hippocampus. Here the quantification showed that the number of LRP1-expressing recombined cells was significantly decreased in the knockout compared to the control **(C)**. Furthermore, the knockout induction was evaluated via RT-PCR **(D)**. The gene expression of *Lrp1* was not altered in LRP1-deficient hippocampi during development compared to control tissue **(E)**. As a last approach the protein expression of LRP1 was analyzed with western blot analysis **(F)**. The protein expression was comparable in both conditions during development **(G)** (Scale bar is 50 μm; mean ± SEM; two-way ANOVA with *post-hoc* Bonferroni test; *p < 0.05, **p < 0.01, ***p < 0.001; for *N*-values see Table 2).

We have previously shown that the deletion of *Lrp1* in neural stem and precursor cells leads to an increased number in GFAP-positive astrocytes (Safina et al., 2016) and that the absence of *Lrp1* in astrocytes caused a delayed maturation and an altered neuronal transmission in neurons *in vitro* (Romeo et al., 2021). Here we were interested how the deletion of astroglial *Lrp1* affects the differentiation of astrocytes and the functionality *in vivo*. Therefore, we used the already described triple transgenic mouse line GLAST::CreERT2xLRP1 (Romeo et al., 2021). In this mouse model LRP1 was specifically deleted in GLAST-positive astrocytes and the recombination events were visualized with the expression of the reporter tdTomato. In this study, we used LRP1-deficient animals (GLAST^CreERT2/wt^Rosa26^fl/fl^LRP1^fl/fl^; further referred to as LRP1^fl/fl^ or knockout) and control animals, with expression of tdTomato (GLAST^CreERT2/wt^Rosa26^fl/fl^LRP1^wt/wt^; further referred to as LRP1^wt/wt^ or control). To induce the conditional knockout, tamoxifen (TAM) was applied to lactating mothers. The application of TAM caused release of CreERT2 from HSP90 and translocation to the nucleus, subsequently followed by the excision of loxP-flanked genes, (*Lrp1* and the stop codon upstream of *Tdtomato* to induce the expression of the reporter) (Weber et al., 2001; Mori and Zhang, 2006). First, we evaluated the recombination efficiency in control and conditional knockout mice at three developmental stages via immunohistochemistry (P21, P28 and adult) within the hippocampus. Therefore, we quantified all tdTomato-positive cells divided by the total number of Hoechst-positive cell nuclei in the molecular layers of the hippocampus. The quantification showed that at P21 92.51% ± 1.73% of the cells in control animals and 94.21% ± 0.36% of the cells in the conditional knockout were recombined (see Figures 1A,B). At P28 the recombination efficiency was similar to P21 (85.48% ± 1.92% in the control mice and 90.97% ± 2.13% in the knockout mice, respectively). The number of recombined cells was stable over time. At the adult stage around 96.77% ± 0.24% of the cells were still tdTomato-positive in LRP1-deficient animals in comparison to 95.4% ± 0.35% in control mice. Next, we investigated the number of LRP1- and tdTomato-double positive cells in both conditions to evaluate the knockout efficiency. Therefore, the number of LRP1- and tdTomato-double positive cells were divided by the number of all tdTomato-positive cells. The expression of LRP1 was significantly decreased in the knockout condition compared to the control condition (P21: LRP1^wt/wt^ 59.76% ± 2.48%, LRP1^fl/fl^ 5.83% ± 1.36%, *p* ≤ 0.0001; P28: LRP1^wt/wt^ 71.05% ± 3.8%, LRP1^fl/fl^ 7.15% ± 1.88%, *p* ≤ 0.0001; adult: LRP1^wt/wt^ 63.45% ± 3.82%, LRP1^fl/fl^ 2.51% ± 0.56%, *p* ≤ 0.0001) at all investigated time points (see Figures 1A,C). To further validate the downregulation of LRP1 in the knockout animals, we performed PCR and western blot analysis. The relative expression of LRP1 was not affected by the knockout induction (see Figures 1D,E). Furthermore, the western blot analysis showed no significant alterations in the protein expression of LRP1 (see Figures 1F,G). However, it needs to be considered that hippocampal tissue was used including other cell types expressing LRP1.

**TABLE 2:** Number of biological N for each performed experiment.

	LRP1^wt/wt^	LRP1^fl/fl^
Immunohistochemistry	P21: *N* = 3 P28: *N* = 4 Adult: *N* = 4	P21: *N* = 5 P28: *N* = 7 Adult: *N* = 6
PCR/western blot	P21: *N* = 3 P28: *N* = 4 Adult: *N* = 3	P21: *N* = 3 P28: *N* = 6 Adult: *N* = 10
Weight	P21: *N* = 6 P28: *N* = 3 Adult: *N* = 9	P21: *N* = 8 P28: *N* = 17 Adult: *N* = 17
Behavioral tests	*N* = 5	*N* = 6

*The number of samples differed between each genotype and developmental timepoint due to the sample preparation.*

**FIGURE 2:**
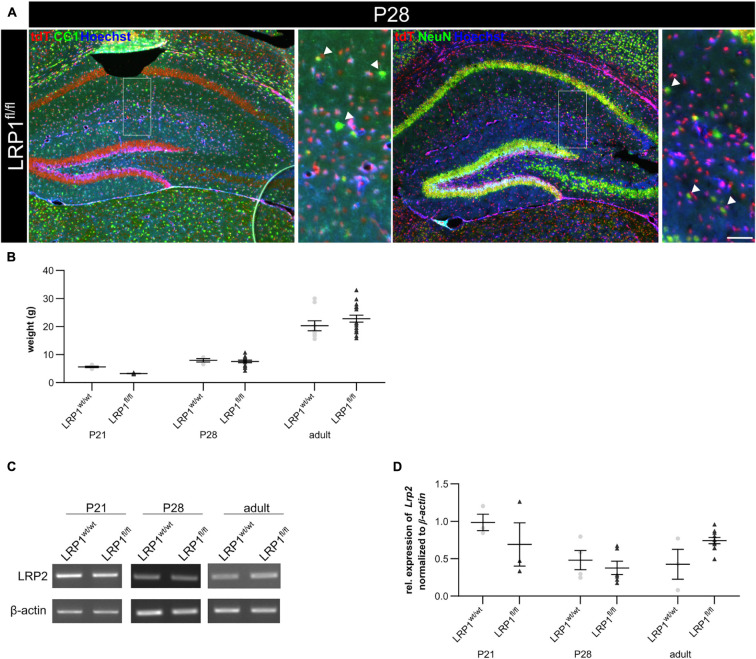
Characterization of the knockout *in vivo*. To confirm that the deletion of LRP1 was restricted to the astrocytic lineage, an immunohistochemical staining against neurons (NeuN; green) and oligodendrocytes (CC1; green) was performed **(A)**. There was no colocalization of both cell-specific markers with the expression of tdTomato in recombined cells indicated by the arrowhead leading to the assumption that the deletion of LRP1 only occurred in astrocytes. To further characterize the newly generated mouse line, the weights of the TAM-receiving animals were compared **(B)**. The statistical analysis revealed no changes in the weight of knockout animals compared to the control. As the deletion of one member of the LDL-family might results in an increased expression of other members, the expression of *Lrp2* **(C,D)** was investigated. However, the knockout induction of LRP1 caused no altered expression of LRP2 (Scale bar is 50 μm; mean ± SEM; two-way ANOVA with *post-hoc* Bonferroni test; for *N*-values see Table 2).

To confirm that the deletion of LRP1 only affected the astroglial lineage an immunohistochemical staining against NeuN and CC1 was performed to exclude neurons and oligodendrocytes. Therefore, knockout tissue of P28 mice was used and almost no co-localization of tdTomato with either NeuN-positive neurons or CC1-positive oligodendrocytes were observed, meaning that the vast majority of cells lacking LRP1 were astrocytes (see Figure 2A). Additionally, we analyzed the weight of LRP1-deficient animals and compared the values to control animals and we saw no difference (see Figure 2B). Due to the similar structure and comparable functions in regard to endocytosis (Auderset et al., 2016), we were interested if the LDL-family member LRP2 was upregulated in hippocampal tissue of LRP1-deficient animals during the development as a compensatory effect. We investigated the expression of the LDL-family member via PCR. The expression of LRP2 was not altered in LRP1-deficient hippocampal tissue in comparison to control tissue (see Figures 2C,D).

### LRP1 Did Not Influence Proliferation of Hippocampal Astrocytes at P21

**FIGURE 3:**
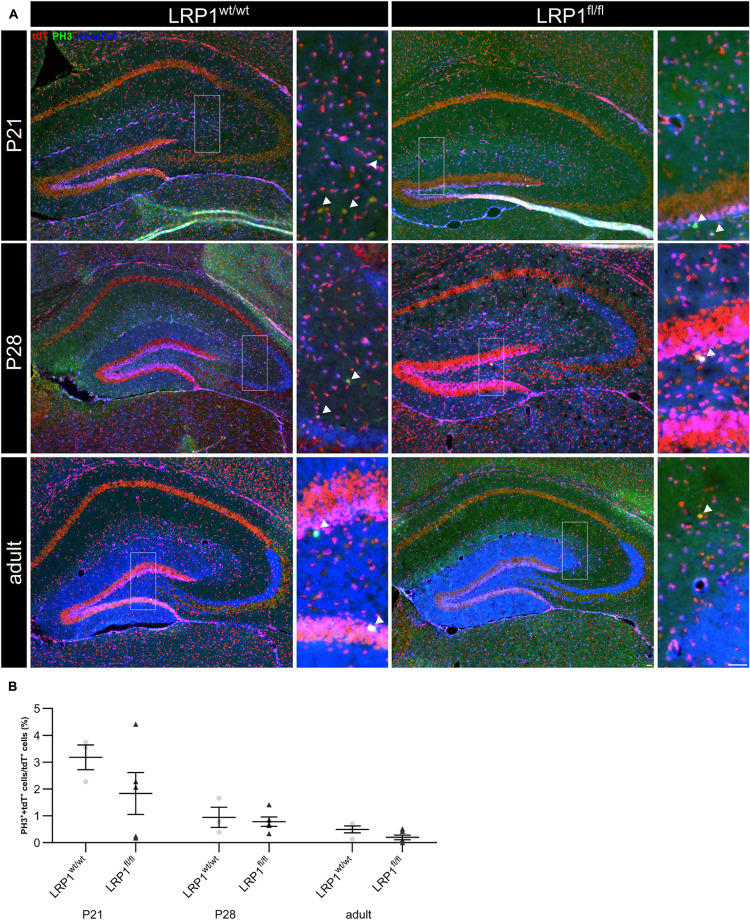
Proliferation capacity of LRP1-deficient astrocytes was not affected. The proliferation rate was evaluated with an immunohistochemical staining against phospho-Histone H3 (PH3; green) and tdTomato (**A**, red). Double-positive cells indicated by the arrowhead were quantified and the analysis showed that the numbers of proliferation events were comparable in both conditions (**B**, Scale bar is 50 μm; mean ± SEM; two-way ANOVA with *post-hoc* Bonferroni test; for *N*-values see Table 2).

Due to the high number of potential ligands LRP1 is known to influence several cellular processes including proliferation. Therefore, we investigated the proliferation capacity of recombined astrocytes via immunohistochemistry (see Figure 3A). The numbers of phospho-Histone H3 (PH3)- and tdTomato-double positive cells were quantified (see Figure 3B). The quantification revealed that the absence of LRP1 caused no altered proliferation rate. Therefore, the number of PH3- and tdTomato-positive cells were comparable in both conditions.

### No Changes in Astroglial Maturation After Loss of LRP1

**FIGURE 4:**
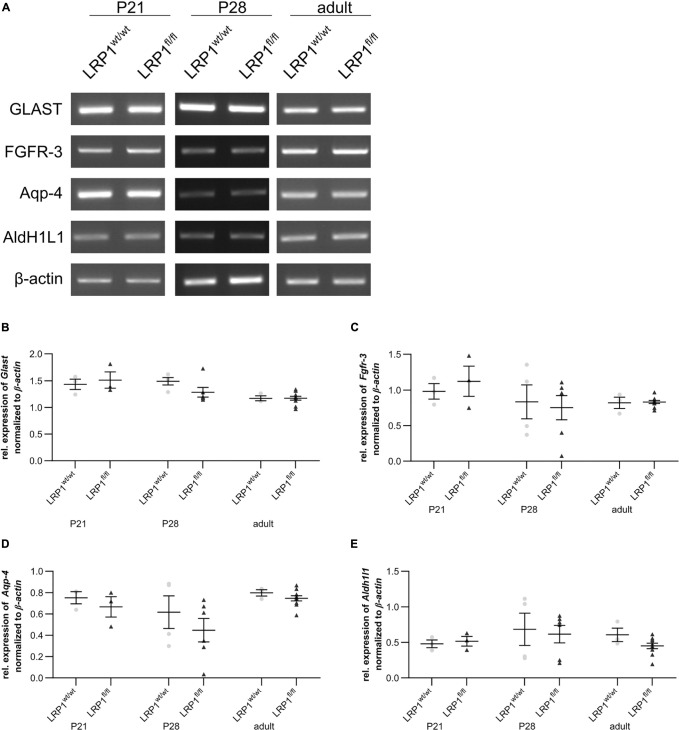
Expression of late precursor or immature astrocytic genes was not altered in LRP1-deficient hippocampi. To investigate the late precursor stage, *Glast* **(A,B)** and *Fgfr-3* **(A,C)** were analyzed, whereas the immature astrocytic stage was examined with the markers *Aqp-4* **(A,D)** and *Aldh1l1* **(A,E)**. The analysis revealed no significant changes in the hippocampal tissue of LRP1-deficient animals compared to control animals during development (Mean ± SEM; two-way ANOVA with *post-hoc* Bonferroni test; for *N*-values see Table 2).

We analyzed whether the deletion of LRP1 had any influence on the gene expression of several markers specific for the late precursor or immature astroglial stage (see Figure 4). To identify late precursor cells favoring an astroglial fate, we used *Glast* (see Figures 4A,B) and *Fgfr-3* (see Figures 4A,C), whereas immature astrocytes were investigated via the expression of *Aqp-4* (see Figures 4A,D) as well as *Aldh1l1* (see Figures 4A,E). The gene expression analysis revealed no changes of any marker in hippocampal tissue of LRP1-deficient animals compared to the control condition, highlighting that LRP1 has no influence on astroglial maturation based on the analyzed markers.

### Decreased Quantity of GFAP-Positive Astrocytes in the Hippocampus at P21

**FIGURE 5:**
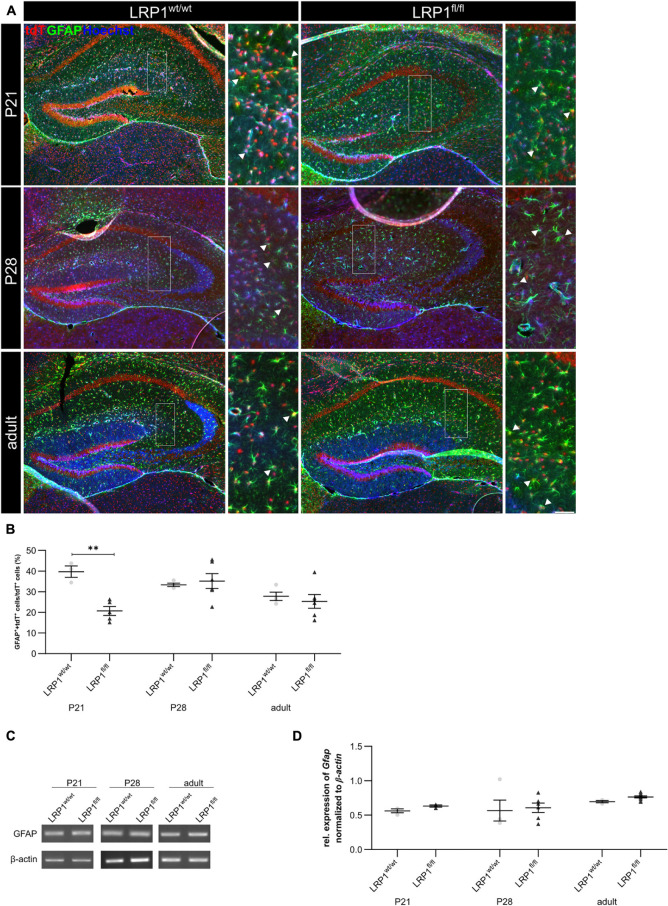
LRP1 depletion in astrocytes resulted in no reactive phenotype. The immunohistochemical staining against GFAP (green) and tdTomato (red) revealed no reactive morphology **(A)**. The quantification of GFAP- and tdTomato-positive cells indicated by the arrowhead showed no changes in the number of double-positive cells in the hippocampi upon LRP1 deletion during development **(B)**. Additionally, RT-PCR analysis was performed to investigate whether the *Gfap* gene expression was altered **(C)**. However, the statistical analysis showed no differences between the knockout and control condition at all three investigated timepoints **(D)** (Scale bar is 50 μm; mean ± SEM; two-way ANOVA with *post-hoc* Bonferroni test; for *N*-values see Table 2).

We previously reported that the deletion of *Lrp1* in neural stem and progenitor cells (NSPCs) caused an increased number of glial fibrillary acidic protein (GFAP)-positive astrocytes (Safina et al., 2016; Bres et al., 2020). However, LRP1-deficient astrocytes did not show a reactive phenotype *in vitro* (Romeo et al., 2021). Via the immunohistochemical staining against GFAP, we wanted to investigate the influence of the LRP1 deletion in regard to a reactive phenotype and in terms of maturation, where GFAP is mainly expressed by mature astrocytes *in vivo* (see Figure 5A). The quantification of GFAP- and tdTomato-double positive cells revealed that the deletion of LRP1 led to a significantly decreased number of double positive cells (19.73% ± 1.68%) in comparison to the control tissue (39.11% ± 2.24%; *p* ≤ 0.01) at P21 (see Figure 5B). With ongoing development, the number of GFAP- and tdTomato-positive astrocytes increased in the knockout mice and was comparable to the control condition reaching adulthood where the numbers stabilized.

Furthermore, mRNA expression of *Gfap* was investigated to support the findings of the immunohistochemical staining (see Figure 5C). Contrary to immunohistochemical data, the mRNA analysis showed that the astroglial loss of *Lrp1* had no effect on *Gfap* mRNA expression at any developmental stage in the hippocampus compared to the control tissue (see Figure 5D).

**FIGURE 6:**
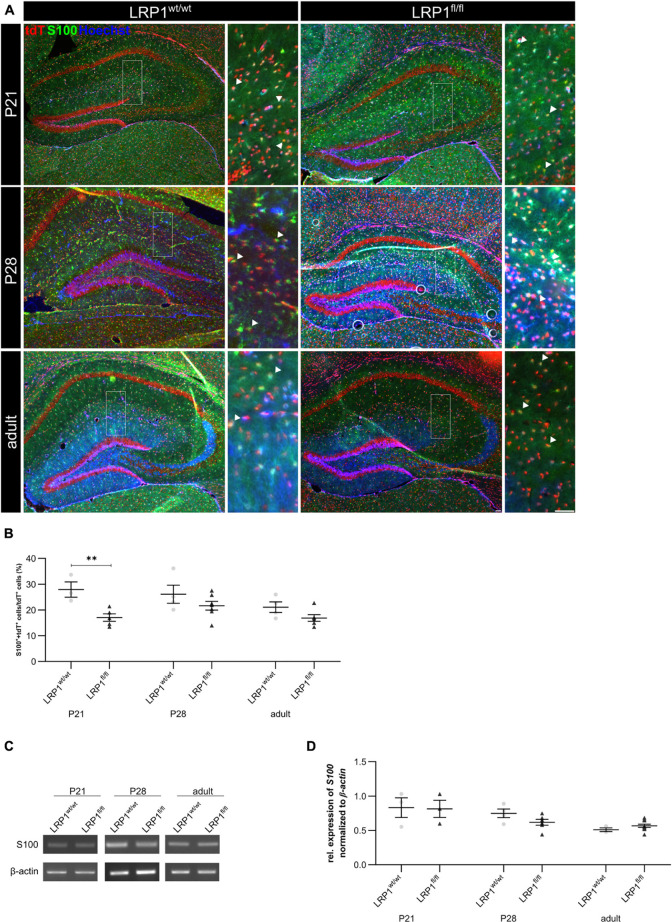
Maturation of LRP1-deficient astrocytes was affected at the beginning of development. To further investigate the maturation of astrocytes upon LRP1 deletion, an immunohistochemical staining against S100 (green) and tdTomato was performed **(A)**. The quantification showed a significantly decreased number of double-positive cells indicated by the arrowhead within the hippocampi of LRP1-deficient animals at P21 compared to the control condition **(B)**. However, with ongoing development the numbers were comparable in both conditions. To support the findings of the staining, RT-PCR analysis was performed to analyze S100 gene expression **(C)**. Nevertheless, the expression was not altered upon LRP1 deletion in hippocampal tissue at all three investigated timepoints **(D)** (Scale bar is 50 μm; mean ± SEM; two-way ANOVA with *post-hoc* Bonferroni test; for *N*-values see Table 2).

Additionally, the number of S100 calcium binding protein β (S100)-positive cells was evaluated to investigate the maturation of LRP1-depleted astrocytes (see Figure 6A). The immunohistochemical staining showed that the number of S100- and tdTomato-positive cells was significantly downregulated in LRP1-deficient hippocampi (16.55% ± 2.88%) when compared to control tissue (28.45% ± 2.44%; *p* ≤ 0.05) at P21 (see Figure 6B). The number of double positive cells was comparable in control and LRP1-deficient mice with ongoing development. However, similar to GFAP, the mRNA expression of *S100* was not affected by the deletion of LRP1 in astrocytes (see Figures 6C,D).

The data indicates that LRP1 mainly influences the maturation of astrocytes in early development in the hippocampus. However, these differences were compensated with increasing age of the animals. Additionally, the regulation by the knockout induction occurred mainly on protein level as the mRNA expression was not affected by the absence of LRP1, contrary to the number of marker-positive cells.

### Expression of Glutamate Transporter GLT-1 Was Not Altered in Astrocytes Upon LRP1 Deletion

**FIGURE 7:**
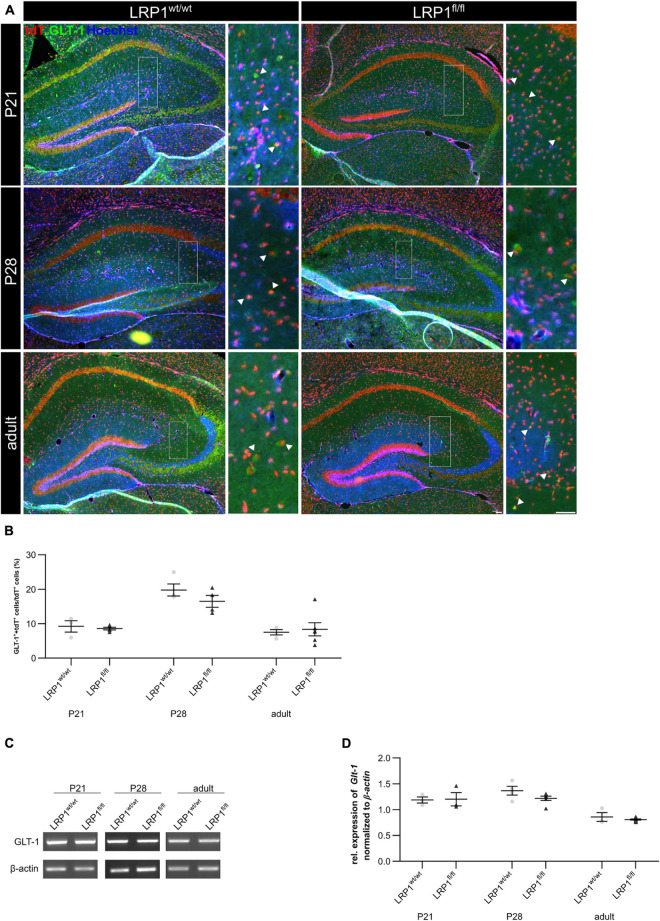
Glutamate transporter expression was not influenced by the deletion of LRP1. As a next approach, the number of GLT-1 (green) and tdTomato-expressing (red) cells was analyzed **(A)**. The statistical evaluation showed no alterations in the number of double-positive cells indicated by the arrowhead in LRP1-depleted tissue compared to the control condition during development **(B)**. Also, the gene expression of *Glt-1* was not affected by the deletion of LRP1 in hippocampal tissue **(C,D)** (Scale bar is 50 μm; mean ± SEM; two-way ANOVA with *post-hoc* Bonferroni test; for *N*-values see Table 2).

Astrocytes have diverse functions and can contribute to different cellular processes. They are involved in synaptic transmission via the uptake of neurotransmitters, like glutamate, out of the synaptic cleft. Therefore, the expression of the glutamate transporter 1 (GLT-1) on the cell surface of LRP1-deficient astrocytes was investigated (see Figure 7A). The quantification of the immunohistochemical staining revealed no alterations in the number of GLT-1- and tdTomato-positive cells in early development at P21 (see Figure 7B). With increasing age, GLT-1 becomes the main glutamate transporter and the number of GLT-1-positive astrocytes should increase. Our analysis showed that the number of GLT-1- and tdTomato-positive cells increased in controls and LRP1-deficient mice but slightly decreased reaching adulthood. Although, no changes in *Glt-1* mRNA expression were observed (see Figure 7C). The analysis showed no alterations in the expression of *Glt-1* in LRP1-deficient hippocampal tissue at all three investigated timepoints in comparison to the control tissue (see Figure 7D). Furthermore, the protein expression of GLT-1 was not affected by the depletion of LRP1 as well (see Supplementary Figures S2A,B).

### LRP1-Deficient Hippocampal Tissue Showed Significantly Decreased Activity of the Ras/Raf/MEK/Erk-Signaling Pathway at P21

**FIGURE 8:**
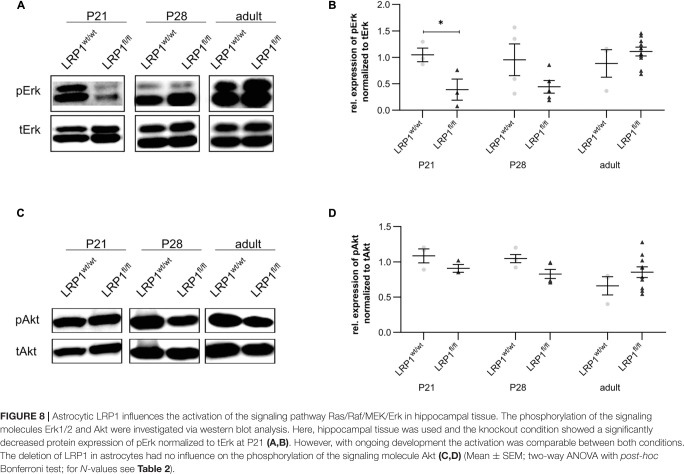
Astrocytic LRP1 influences the activation of the signaling pathway Ras/Raf/MEK/Erk in hippocampal tissue. The phosphorylation of the signaling molecules Erk1/2 and Akt were investigated via western blot analysis. Here, hippocampal tissue was used and the knockout condition showed a significantly decreased protein expression of pErk normalized to tErk at P21 **(A,B)**. However, with ongoing development the activation was comparable between both conditions. The deletion of LRP1 in astrocytes had no influence on the phosphorylation of the signaling molecule Akt **(C,D)** (Mean ± SEM; two-way ANOVA with *post-hoc* Bonferroni test; for *N*-values see Table 2).

The deletion of LRP1 in astrocytes mainly influenced the maturation at an early developmental timepoint. As previously described the maturation of glial cells is dependent on the Ras/Raf/MEK/Erk and PI3K/Akt signaling cascade. Therefore, we were interested whether the phosphorylation of the signaling molecules Erk1/2 and Akt were influenced by the deletion of LRP1 in astrocytes and therefore might explain the delayed maturation we observed at P21 (see Figure 8). The western blot analysis showed a significant downregulation of the active phospho-Erk (pErk) in P21 hippocampal tissue after astroglial loss of LRP1 (0.418 ± 0.197) compared to controls (1.138 ± 0092; *p* ≤ 0.05) (see Figures 8A,B). However, with ongoing maturation the protein expression was comparable in both conditions. The activation of pErk in astrocytes was strongly decreased as we investigated hippocampal tissue including other cell types, also expressing several signaling molecules, such as pErk.

The expression of pAkt was not influenced by the deletion of LRP1 in astrocytes in comparison to the controls at any investigated timepoint (see Figures 8C,D) as we could find no alterations in the gene expression of *Akt* and *Mtor* after loss of LRP1 (data not shown).

### Deletion of Astrocytic LRP1 Negatively Influenced Activity of Hippocampal Neurons *in vivo*

**FIGURE 9:**
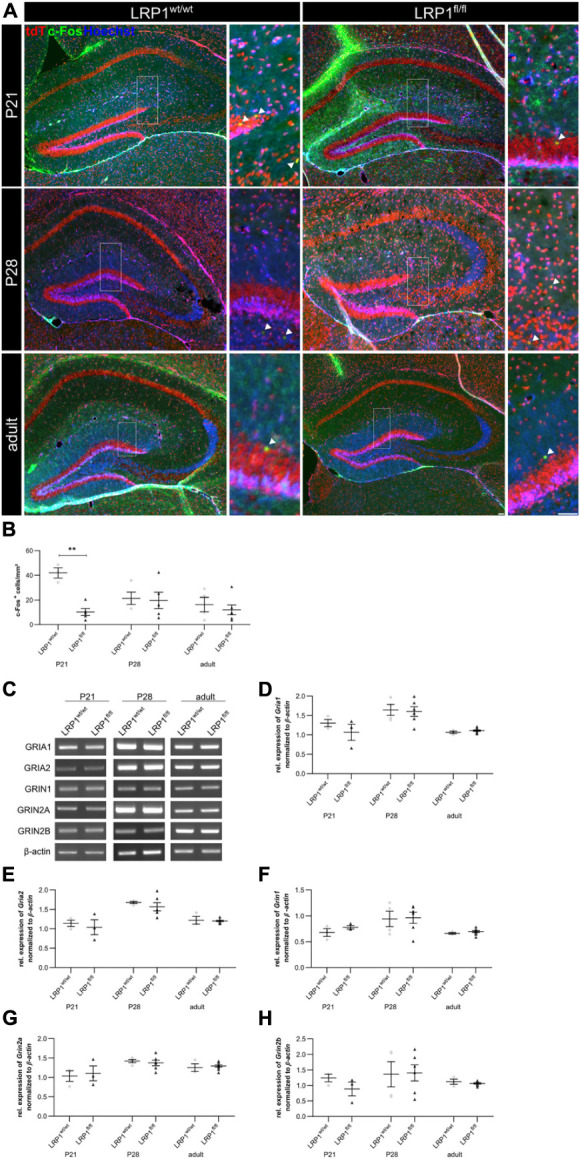
Neuronal activity was negatively influenced in the knockout condition. To assess the neuronal activity within the hippocampus of LRP1-deficient animals, an immunohistochemical staining against c-Fos (green) and tdTomato (red) was performed **(A)**. The quantification of all c-Fos-positive cells indicated by the arrowhead within the hippocampus divided by the area of the hippocampus revealed a significantly decreased number of activated neurons in the knockout condition at P21 compared to the control **(B)**. However, with ongoing maturation the differences were compensated. To further validate the effect of astrocytic LRP1 on the neuronal activity RT-PCR was performed **(C)**. The expression of AMPAR subunits *Gria1* **(D)** and *Gria2* **(E)** was not altered in hippocampal knockout tissue. Furthermore, the NMDAR subunits *Grin1* **(F)**, *Grin2a* **(G)** and *Grin2b* **(H)** also showed no altered expression profile upon the deletion of LRP1 during development (Scale bar is 50 μm; mean ± SEM; two-way ANOVA with *post-hoc* Bonferroni test; *p < 0.05, **p < 0.01, ***p < 0.001; for *N*-values see Table 2).

To further investigate the effect of LRP1 loss in astrocytes to hippocampal tissue, we investigated the activity of neurons via the staining against c-Fos, marking activated neurons (see Figure 9A). Thereby, we deduced whether the metabolic supply of neurons via astrocytes was influenced by the deletion of *Lrp1*. All c-Fos-positive cells were quantified and the number of positive cells per mm^2^ was measured (see Figure 9B). The quantification showed a significantly decreased number of c-Fos-positive neurons after loss of LRP1 (10.53 cells/mm^2^ ± 3.15 cells/mm^2^) when compared to the control condition (41.90 cells/mm^2^ ± 3.41 cells/mm^2^; *p* ≤ 0.01) at P21. With ongoing maturation, the number of c-Fos-positive neurons were similar in both conditions.

Furthermore, the expression of subunits of the glutamate receptors α-amino-3-hydroxy-5-methyl-4-isoxazolepropionic acid (AMPA) and N-methyl-D-aspartate (NMDA) were investigated via RT-PCR (Figure 9C). We investigated the gene expression of the AMPAR subunits *Gria1* and *Gria2* (see Figures 9D,E) as well as the NMDAR subunits *Grin1*, *Grin2a*, and *Grin2b* (see Figures 9F–H) as it was previously proposed that these subunits can be influenced via LRP1. However, the expression analysis revealed no alterations in the gene expression of the receptor subunits in LRP1-deficient hippocampal tissue compared to the control during development.

### Deletion of Astrocytic LRP1 Had No Influence on Motor Coordination or Cognitive Abilities in Adult Animals

**FIGURE 10:**
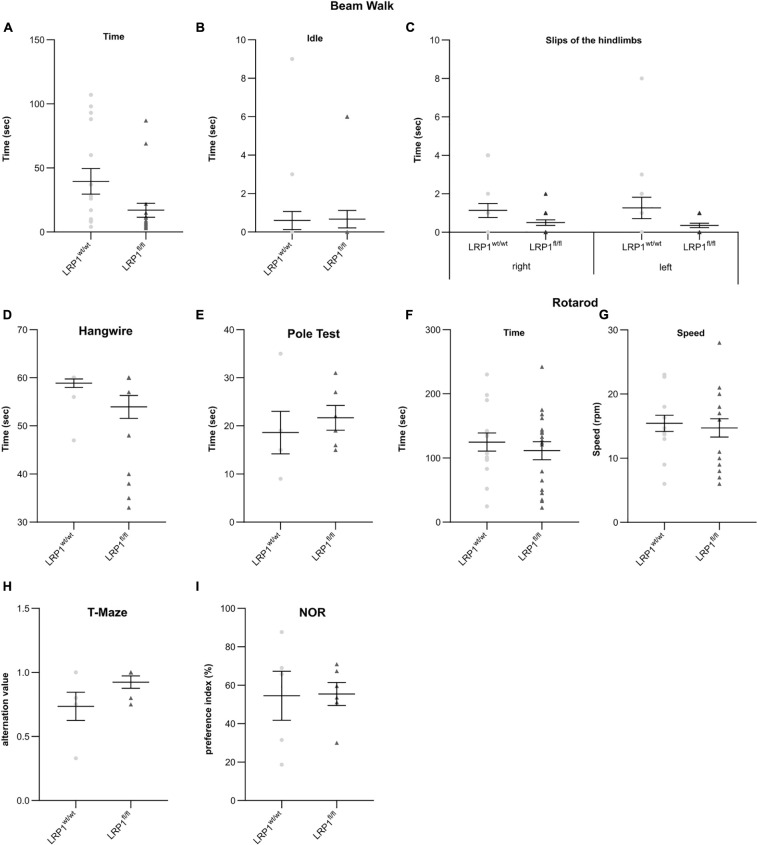
LRP1-deficient animals showed no motor or cognitive disabilities in adulthood. For the further characterization of the newly generated mouse model several motor coordination and cognitive tests were performed. The beam walk test **(A–C)** as well as the pole test **(E)** assessed fine motor coordination and balance whereas the hangwire test **(D)** evaluates strength with little coordination. As a last test, the rotarod test was used **(F,G)** to investigate endurance and balance. All tests were performed with adult animals and LRP1-deficient animals showed to motor coordination disabilities compared to control animals. Additionally, the T-maze test **(H)** and novel object recognition test (NOR; **I**) were used to highlight cognitive abilities and again LRP1-deficient animals behaved the same as control animals (mean ± SEM; Student’s *t*-test; for *N*-values see Table 2).

To assess if the astrocytic deletion of LRP1 had any influence of the behavior or the motor coordination on adult knockout animals several tests were performed. Fine motor coordination and balance were investigated with the beam walk test. The first investigated parameter was the time that was needed by the animals to cross the beam (see Figure 10A), the second parameter was the time left immobile until they started walking on the beam for the first time (see Figure 10B) and the last parameter were the slips of the hindlimbs, either right or left, of the animals while crossing the beam (see Figure 10C). In general, the LRP1-deficient animals exhibited no motor dysfunctions or imbalance in comparison to the control animals. However, the number of slips of the hindlimbs were slightly decreased compared to the control animals, hinting an increased balance. Next, we used the hangwire test to evaluate strength with little coordination. The knockout animals performed in the same way as the control animals in this task (see Figure 10D). Additionally, the motor coordination and balance were analyzed with the pole test. The time needed to descend down to the cage was not altered in the knockout group compared to control animals (see Figure 10E). The last motor coordination test was the rotarod. The investigated parameters here were the time when the animals fell down (see Figure 10F) as well as the speed of the rod when the animals fell down (see Figure 10G). Again, we saw no differences in the motor coordination of LRP1-depleted animals.

The cognitive abilities of the LRP1-deficient animals were assessed with the T-maze test (see Figure 10H) and the novel object recognition (NOR) test (see Figure 10I). These two tests revealed that the knockout animals showed no cognitive disabilities in comparison to the control group.

These findings lead to the assumption that the astrocytic deletion of LRP1 had no effect on the motor coordination as well as on the cognitive abilities of adult animals.

## Discussion

We used the newly generated mouse model with an inducible loss of astroglial LRP1 to examine how the maturation and maintenance of astrocytes is influenced by astroglial LRP1. Therefore, we decided to investigate the maturation on protein (immunohistochemistry, western blot) and mRNA level (RT-PCR). The application of Tamoxifen caused a significantly decreased number of LRP1-expressing cells in the knockout condition compared to the control condition. However, western blot analysis revealed no significant downregulation. Nevertheless, the analysis was performed with hippocampal tissue, including other cell types, such as neurons and microglia, beside the knockout-affected astrocytes. Previous studies showed that 50% of LRP1 protein is expressed by neurons (Shinohara et al., 2017; Liu et al., 2019), possibly masking the deletion of LRP1 in astrocytes. The further analysis of the knockout specificity revealed that only negligible amount of either NeuN- or CC1-positive cells were expressing tdTomato. However, previous studies reported that CC1 was detectable in a small proportion of astrocytes (Bhat et al., 1996; Fuss et al., 2000; Mori et al., 2006; Bin et al., 2016). Mori et al. (2005) have reviewed the concurring expression of astrocyte-specific markers also in radial glia cells. Recombination under the GFAP promotor was detectable in neurons derived from radial glia cells (Malatesta et al., 2003). Leading to the assumption that tdTomato- and NeuN-double positive cells observed in the described animal model might resemble newborn neurons derived from radial glia cells located in the dentate gyrus affected by the recombination. However, as neither neurons nor oligodendrocytes are expressing the used astrocyte-specific markers, the analysis performed in this study resembled the effect of LRP1 on the maturation of astrocytes.

After successful confirmation of astroglia-specific LRP1 loss *in vivo*, we investigated the proliferation behavior of recombined cells within the hippocampus during development and observed no changes in astroglial proliferation rate. We previously reported that the proliferation of neural stem and precursor cells *in vitro* was negatively influenced by the deletion of LRP1 (Safina et al., 2016). However, the role of LRP1 in regard to proliferation is opposed, depending on the cell type and tissue.

We tracked the astroglial differentiation with stage-specific markers according to Wiese et al. (2012). However, the gene analysis revealed no significant alterations in regard to the expression of late precursor markers, such as GLAST and FGFR-3, nor to immature astrocytes markers, such as AldH1L1 and Aqp-4. Nevertheless, the protein expression is not always correlating to the mRNA levels, as the relationship between mRNA to protein synthesis is objected to fluctuations caused by different factors, such as resource availability (Liu et al., 2016). Therefore, the number of positive cells might be altered, whereas the mRNA levels were not affected by the LRP1 deletion.

As a next step, we investigated mature astrocytes with immunohistochemistry. Here we used GFAP, a prototypical marker for mature astrocytes and a reliable maker for reactive astrogliosis (Pekny et al., 1995; Pekny and Pekna, 2004; Herrmann et al., 2008). Our analysis showed a significantly decreased number of recombined cells expressing GFAP at P21 in knockout mice compared to controls. Barcelona et al. (2011) showed that the expression of GFAP in retinal Müller glia was induced by application of α2-macroglobulin. This effect was abolished by using the LRP1 inhibitor RAP. Therefore, the binding of α2-macroglobulin to LRP1 facilitates the expression of GFAP. The deletion of LRP1 in astroglia might cause the same effect, leading to a decreased number of GFAP-positive astrocytes observed in our animal model. Furthermore, we assessed mature astrocytes with S100 and again the number of tdTomato- and S100-double positive cells was significantly decreased in the hippocampus at P21. However, the number of GLT-1- and tdTomato-double positive cells was not affected by the LRP1-knockout. Nevertheless, we observed a decreased number of GLT-1- and tdTomato-double positive cells in both conditions reaching adulthood. Benediktsson et al. (2012) observed a correlation between the number of GLT-1-positive astrocytes and neuronal activity. Therefore, we hypothesized that the observed decreased neuronal activity in the adult stage resulted in a reduced number of GLT-1- and tdTomato-positive cells in both conditions.

Our results indicate that LRP1 is mainly essential for astroglial maturation during an early developmental phase causing a decreased maturation at this timepoint, which is compensated with ongoing development. We hypothesized that this effect might be dependent on the activation of signaling cascades as it is known that LRP1 can form complexes with other receptors on the cell surface, which induce downstream activation of signaling cascades (Seger and Krebs, 1995; Zhang and Liu, 2002; Ortega and Alcántara, 2010; Burris, 2013; Kumar et al., 2013).

Previous studies already reported that the postnatal proliferation and terminal differentiation of glial cells is dependent on cell intrinsic cues (Qian et al., 2000; Shen et al., 2006). The Ras/Raf/MEK/Erk pathway was described to be critical for the glial specification as well as for proliferation (Li et al., 2012). The activation of this signaling cascade can be induced by the complex formation of LRP1 with PDGFRβ (Boucher et al., 2002; Loukinova et al., 2002) or with trkA (Spuch et al., 2012). Therefore, we examined the phosphorylation and therewith the activation of signaling molecules Erk1/2 and Akt. Our analysis showed a significantly downregulated phosphorylation of Erk1/2 in hippocampal tissue of P21 knockout animals, which correlates to the delayed maturation observed at the same developmental stage. Therefore, we conclude that LRP1 is a regulator of the early astrocytic maturation by influencing the activation of the Ras/Raf/MEK/Erk pathway, while the maintenance of astroglial numbers seems to be not influenced by the loss of LRP1.

Based on our findings, we were interested if the delayed maturation of astrocytes had any influence on the activity of neurons as we previously observed that LRP1-depleted astrocytes negatively influenced the activity of hippocampal neurons in a model of the tripartite synapse *in vitro* (Romeo et al., 2021). We used c-Fos to investigate the number of activated neurons within the hippocampus and saw a significantly decreased number of c-Fos-positive cells at P21, which were not accompanied by an altered expression of NMDA or AMPA receptor subunits. This result corresponds to the delayed maturation of astrocytes at the same timepoint. Previous studies showed that the neuronal differentiation as well as neurite outgrowth is dependent on the activation of the Ras/Raf/MEK/Erk pathway induced via astrocytes as it promoted the astrocytic release of laminin (Spohr et al., 2011, 2014). We observed a decreased phosphorylation of the signaling molecule Erk1/2 involved in this signaling pathway, which besides negatively influencing the astroglial maturation also caused an altered neuronal differentiation or neurite outgrowth ultimately resulting in a decreased neuronal activity observed in our model. Further studies might highlight if the observed decreased phosphorylation of Erk1/2 is facilitated via the binding of LRP1 ligands, such as tPA or α2-macroglobulin, or the interaction of LRP1 with other receptors, like integrins or PDGFR.

The altered neuronal activity observed in our animal model might also result from other factors, as astrocytes are tightly associated to neuronal synapses by forming the tripartite synapse. Here, astrocytes are able to release gliotransmitters, such as GABA, ATP, and D-serine, directly influencing the activity of the pre- or postsynapse (Araque et al., 1999; Volterra and Steinhäuser, 2004; Perea et al., 2009). Another neuromodulating factor released by astrocytes is the tissue plasminogen activator (tPA) (Fernández-Monreal et al., 2004; Samson and Medcalf, 2006). It was previously reported that the inhibition of LRP1 via RAP caused a decreased uptake of tPA out of the synaptic cleft facilitated via astrocytes (Cassé et al., 2012). Thereby, astrocytes cannot buffer the potentiated NMDA receptor activity by tPA, ultimately resulting in neurotoxicity due to high levels of glutamate within the synaptic cleft (Cassé et al., 2012; Jeanneret et al., 2016).

To further characterize our new mouse model, we performed several motor coordination and cognitive tests with adult animals. However, the LRP1-deficient animals showed no disabilities or cognitive restrictions in comparison to control animals of the same age.

It is of interest to compare the outcome of the present investigation with results obtained by deleting LRP1 from cortical radial glia stem cells using the Emx1-Cre driver line (Bres et al., 2020). In that model, recombination started at E9.5 and affected both cortex and hippocampus, targeting neurogenesis as well as gliogenesis. The resulting mouse line displayed severe neurological symptoms, expanded ventricles and seizures. As a consequence, astrocytes became reactive starting at P21 and up-regulated GFAP. Unexpectedly, however, the hippocampus at P28 exhibited a reduction of c-fos-positive cells, comparable to the current study. In the present approach, LRP1 was deleted from the astrocyte lineage during the postnatal period, after the phase of neurogenesis had been completed. As consequence, astrocyte maturation in the hippocampus was delayed and neuronal activity in the hippocampus appeared reduced at P14, as judged from c-fos expression. We conclude that elimination of LRP1 from the astrocyte lineage *per se* is not sufficient to explain the hyperactive phenotype observed in our previous study. In this our model is in agreement with a recent report where LRP1 had been removed using a GFAP-driven Cre-recombinase (Liu et al., 2017).

Summarized our results showed that the deletion of LRP1 in astrocytes resulted in a delayed maturation at an early developmental stage, accompanied by a decreased activation of signaling molecules. However, the gene expression of any stage-specific marker related to the astrocytic lineage was not influenced by the knockout induction. Though, the immunohistochemical analysis revealed a decreased number of mature astrocytes. Additionally, the neuronal activity was negatively influenced by the deletion of astroglial LRP1 at the same developmental stage. Nevertheless, the observed effects were compensated with ongoing development resulting in comparable cell numbers and no motor or cognitive deficits in adult conditional knockout mice.

## Conclusion

In conclusion, we could identify LRP1 as a novel regulator of the early astroglial differentiation in the hippocampus by promoting the phosphorylation of Erk1/2 involved in the Ras/Raf/MEK/Erk signaling pathway.

## Data Availability Statement

The raw data supporting the conclusions of this article will be made available by the authors, without undue reservation.

## Ethics Statement

The animal study was reviewed and approved by the State Agency for Nature, Environment and Consumer Protection Northrhine-Westphalia (Landesamt fuer Umweltschutz, Naturschutz und Verbraucherschutz; file number: 84-02.04.2016.A482). Written informed consent was obtained from the owners for the participation of their animals in this study.

## Author Contributions

RR performed the experiments regarding the transcardial perfusion, sample preparation, immunohistochemistry, as well as RT-PCR. RR and AF wrote and revised the manuscript. DM and MM performed the behavioral and cognitive tests. MM and AS revised the manuscript. All authors contributed to the article and approved the submitted version.

## Conflict of Interest

The authors declare that the research was conducted in the absence of any commercial or financial relationships that could be construed as a potential conflict of interest.

## Supplementary Material

Supplementary Figure 1 Exemplary picture of analyzed area. The quantification of recombined and marker-positive cells occurred in the spaces between the *Cornu Ammonis* layers of the hippocampus. Here, the vast majority of cells were tdTomato-positive, leading to the assumption that the vast majority were astroglia.

Supplementary Figure 2 Protein expression of GLT-1 was not altered upon knockout induction. The deletion of LRP1 from astrocytes did not cause any changes in the protein expression of GLT-1 (**A,B**; mean ± SEM; two-way ANOVA with *post-hoc* Bonferroni test; for *N*-values see Table 2).
