# Supplementary figures and images for: Low-Density Lipoprotein Receptor-Related Protein 1 (LRP1) as a Novel Regulator of Early Astroglial Differentiation
Source: Front Cell Neurosci. 2021 Feb 18;15:642521. doi: 10.3389/fncel.2021.642521 (PMC7930235; doi:10.3389/fncel.2021.642521)

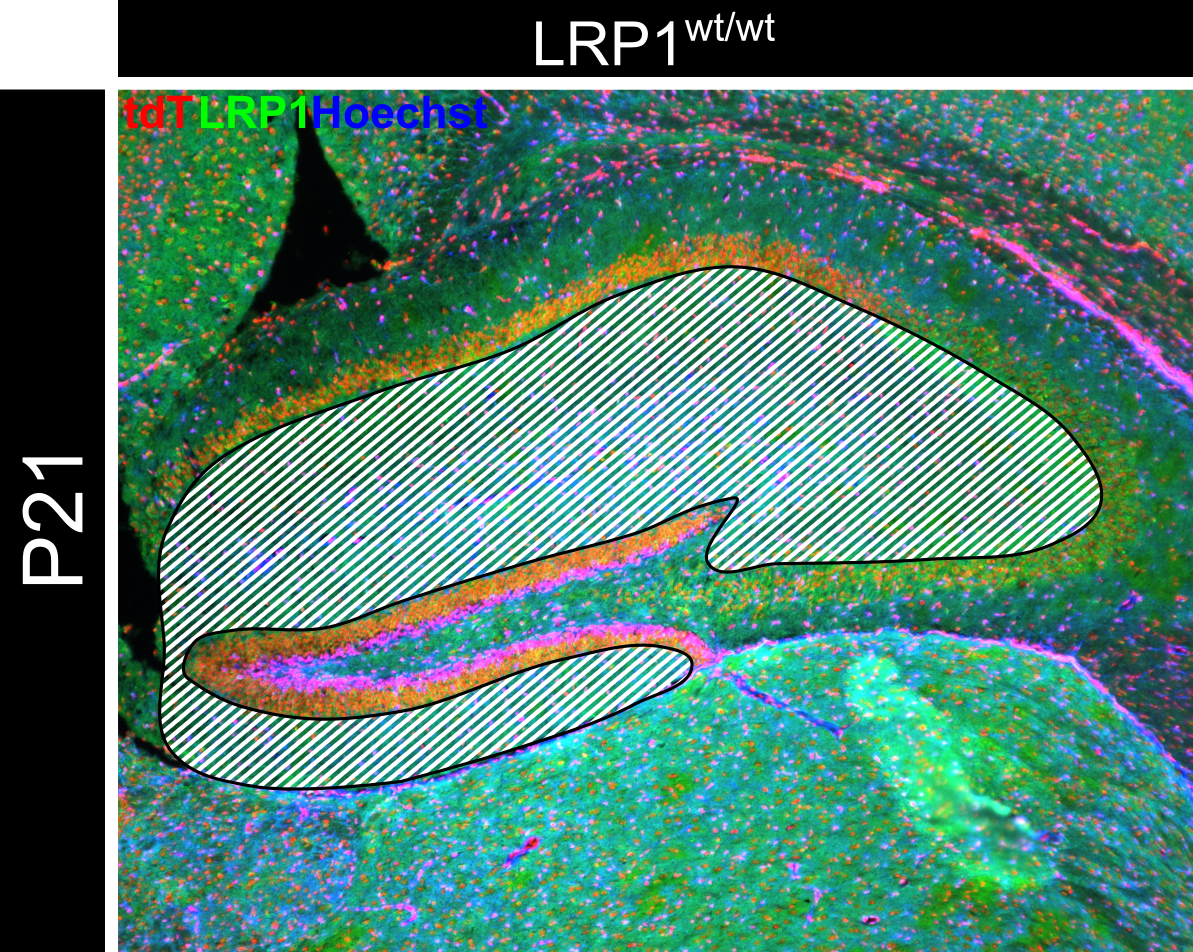

Supplement: Supplementary Figure 1 — Exemplary picture of analyzed area. The quantification of recombined and marker-positive cells occurred in the spaces between the Cornu Ammonis layers of the hippocampus. Here, the vast majority of cells were tdTomato-positive, leading to the assumption that the vast majority were astroglia. [file Image_1.TIF]

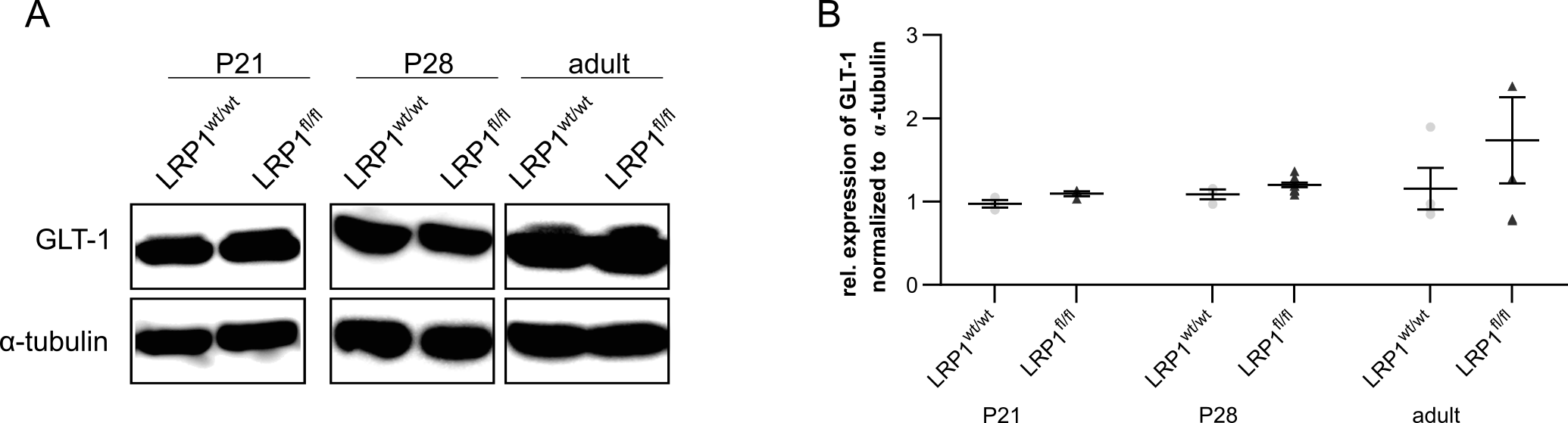

Supplement: Supplementary Figure 2 — Protein expression of GLT-1 was not altered upon knockout induction. The deletion of LRP1 from astrocytes did not cause any changes in the protein expression of GLT-1 (A,B; mean ± SEM; two-way ANOVA with post-hoc Bonferroni test; for N-values see Table 2). [file Image_2.TIF]
